# Optimal response to dimethyl fumarate is mediated by a reduction of Th1‐like Th17 cells after 3 months of treatment

**DOI:** 10.1111/cns.13142

**Published:** 2019-05-07

**Authors:** María José Mansilla, Juan Navarro‐Barriuso, Silvia Presas‐Rodríguez, Aina Teniente‐Serra, Bibiana Quirant‐Sánchez, Cristina Ramo‐Tello, Eva María Martínez‐Cáceres

**Affiliations:** ^1^ Immunology Division, LCMN Germans Trias i Pujol University Hospital and Research Institute, Campus Can Ruti Badalona Spain; ^2^ Department of Cellular Biology, Physiology and Immunology Universitat Autònoma de Barcelona Cerdanyola del Vallès Spain; ^3^ Multiple Sclerosis Unit, Department of Neurosciences Germans Trias i Pujol University Hospital Badalona Spain; ^4^ Department of Medicine Universitat Autònoma de Barcelona Cerdanyola del Vallès Spain

**Keywords:** biomarkers, dimethyl fumarate, immunomonitoring, multiple sclerosis, Th1‐like Th17 lymphocytes

## Abstract

**Aim:**

Dimethyl fumarate (DMF) is one of the most promising therapies for relapsing‐remitting multiple sclerosis (RRMS) patients since it has shown immunomodulatory and neuroprotective effects. However, a percentage of RRMS patients do not exhibit an optimal response to DMF. The objective of this study was to identify early biomarkers of treatment response by analyzing changes in peripheral leukocyte subpopulations directly in whole blood samples.

**Methods:**

A longitudinal and prospective study analyzing peripheral blood leukocyte subpopulations in 22 RRMS patients before initiating DMF treatment (baseline) and at 1, 3, 6, and 12 months of follow‐up was performed. Differences between no evidence of disease activity (NEDA) and ongoing disease activity (ODA) patients were analyzed.

**Results:**

The beneficial effect of DMF was associated with a specific depletion of memory CD4^+^ and CD8^+^ T lymphocytes and B cells. Importantly, only NEDA patients showed (a) a shift from a pro‐ to an antiinflammatory profile, with an increase of Th2 cells and a decrease of Th1‐like Th17 lymphocytes; and (b) an increase of regulatory CD56^bright^ NK cells.

**Conclusion:**

The optimal response to DMF is mediated by a shift to antiinflammatory and immunoregulatory profile, which puts forward Th1‐like Th17 lymphocytes as a potential early biomarker of treatment response.

## 1 INTRODUCTION

Multiple sclerosis (MS) is a chronic immune‐mediated disease characterized by inflammation, demyelination, and axonal damage in the central nervous system (CNS). During the last years, numerous new immunomodulatory drugs have been approved for the treatment of MS patients. From them, delayed‐release dimethyl fumarate (DMF) is one of the most promising new treatments for relapsing‐remitting MS (RRMS) patients, since it has showed both antiinflammatory and neuroprotective properties. Clinically, DMF treatment reduces the annualized relapse rate (ARR) and the number of new lesions measured by magnetic resonance imaging (MRI).^1^, ^2^

Nowadays, due to the numerous disease‐modifying therapies (DMT) available for MS patients, the selection of the proper treatment for each patient is getting more difficult. Currently, the decision is based on the balance between the potential clinical benefits and the adverse effects of each treatment. However, it is known that a percentage of patients exhibit a suboptimal clinical response to some DMT. Research groups around the world have been investigating the mechanism of action of each DMT, trying to identify molecules or biomarkers of treatment response. Dimethyl fumarate mediates immunomodulatory as well as antioxidative and cytoprotective effects through the inhibition of the transcription factor nuclear factor kappa b (NF‐κB) and promoting the activation of the nuclear factor‐erythroid 2‐related factor (Nfr2) transcriptional pathway.^3^, ^4^, ^5^, ^6^

Studies focused on the mechanism of action of DMF demonstrated that the treatment induces lymphopenia, selectively depleting memory T and B cells and promoting a shift toward a tolerogenic immune profile.^7^, ^8^, ^9^, ^10^, ^11^, ^12^ Specifically, Medina and colleagues reported that DMF induces a change in the ratio between regulatory CD56^bright^ NK cells and effector TNF‐α‐producing CD8^+^ T cells, which is required for the efficacy of the treatment. The authors also suggested that monitorization of leukocyte subpopulations could be a useful tool for early identification of responder and nonresponder patients.^12^

In this study, we performed a longitudinal and extensive characterization of whole blood immunophenotype of RRMS patients before and after 1, 3, 6, and 12 months of DMF treatment by flow cytometry. Changes in lymphocyte subpopulations in patients with no evidence of disease activity (NEDA) in comparison with patients with ongoing disease activity (ODA) were analyzed in order to identify early biomarkers of treatment response. This technique is an easy and fast way to directly analyze (without the limitations of leukocyte isolation or cryopreservation) the *ex vivo* effect of DMF or other treatments in leukocyte subpopulations. Using this protocol, we identified that a reduction of effector memory Th1‐like Th17 cells is associated with increased DMF efficacy, which occurs already after 3 months of treatment in NEDA patients and it is maintained until 12 months of treatment.

## 2 PATIENTS AND METHODS

### 2.1 Patients and study design

A total of 22 RRMS patients from the MS unit of the Germans Trias i Pujol Hospital (Spain), fulfilling McDonald's 2010 criteria, were included in the study before initiating DMF treatment. Clinical, radiological, and immunological monitoring of patients was conducted from baseline (before treatment) until 12 months of treatment. Clinical and demographic characteristics of the patients are summarized in Table 1.

**Table 1:** Clinical and demographic characteristics of multiple sclerosis patients

	Total MS patients	NEDA	ODA
n	22	15	6
Age at baseline	36.7 ± 7.42	38.0 ± 7.53	33.8 ± 7.47
Gender (% female)	15/22 (68.2%)	11/15 (73.3%)	3/6 (50.0%)
Disease duration (y)	4.18 ± 4.72	4.60 ± 5.12	3.67 ± 4.13
Prior treatment			
Naïve	15	11	3
IFN‐β	4	3	1
Fingolimod	1	–	1
Aubagio	1	–	1
Others^a^	1	1	–
Baseline ARR	1.14 ± 0.62	0.99 ± 0.48	1.39 ± 0.83
Relapses during the previous year	1.50 ± 0.91	1.33 ± 0.90	1.83 ± 0.98
Relapses after 12 mo of DMF treatment	0.15 ± 0.37	–	0.50 ± 0.55
Baseline EDSS	2.34 ± 1.47	2.27 ± 1.58	2.75 ± 1.21
EDSS after 12 mo of DMF treatment	2.26 ± 1.93	2.04 ± 1.84	3.17 ± 1.97
Baseline T2 lesions			
<9 T2 lesions	5/22 (22.7%)	4/15 (26.7%)	1/5 (20.0%)
>9 T2 lesions	17/22 (77.3%)	11/15 (73.3%)	5/6 (80.0%)
New T2 lesions after 12 mo of treatment (%)	4/21 (19.04%)	–	4/6 (66.7%)
Presence of gadolinium‐enhancing lesions at baseline	10/21 (47.6%)	6/15 (40.0%)	4/6 (66.7%)
New gadolinium‐enhancing lesions after 12 mo of DMF treatment	1/21 (0.05%)	–	1/6 (16.7%)

Abbreviations: ARR: annual relapse rate; EDSS: Expanded Disability Status Scale; NEDA: no evidence of disease activity; ODA: ongoing disease activity.

a Others: cell therapy clinical trial.

Clinical data included disability progression (defined as worsening of 1 point or more on the Expanded Disability Status Scale [EDSS] score) and disease relapses (defined as new or worsening neurological deficits lasting 24 hour or more in the absence of fever or infection) after 12 months of treatment.

Radiological activity was measured by determining the presence of new or enlarging T2 lesions and/or gadolinium‐enhancing T1 lesions (Gd+) in brain MRI. Either 1.5 or 3.0 Tesla MRI scans were used to evaluate the number of Gd+ lesions before and after 12 months of follow‐up. The same equipment was applied for each patient. The number of Gd+ lesions was visually determined, comparing the first and second MRI scans.

Patients were classified as NEDA (no evidence of disease activity)—defined as the absence of relapses, disability progression, and radiological activity during the follow‐up—or ODA—when an increase in clinical and/or radiological activity was reported.

Blood samples were obtained from patients at baseline and after 1, 3, 6, and 12 months of treatment, to determine changes in lymphocyte subpopulations related to an optimal clinical response to DMF.

The study protocol was approved by the ethics committee of the Germans Trias i Pujol Hospital, and all the patients gave their informed consent to participate in the study.

### 2.2 Flow cytometry analysis

#### 2.2.1 Analysis of peripheral leukocyte subpopulations

For the analysis of the different peripheral blood leukocyte subpopulations, whole blood samples, obtained by standard venipuncture in EDTA tubes, were kept at room temperature and processed within the next 24 hour. Briefly, 100µl of peripheral blood was incubated for 20 minutes at room temperature and protected from light, with the appropriate combination of antibodies (see below) to analyze T‐cell, Treg, B‐cell, and DC/monocyte/NK‐cell subpopulations. After erythrocyte lysis and cell fixation, samples were washed twice and acquired on a LSR II Fortessa cytometer and analyzed using FACSDiva software (BD Biosciences). Cell markers used to define each leukocyte subpopulations are specified in Table S1. The gating strategy used is based on an international consensus and was previously described by Teniente‐Serra and Quirant‐Sánchez.^13^, ^14^

Leukocyte subpopulations were analyzed by using the following four panels of monoclonal antibodies, previously reported by Quirant‐Sánchez et al^14^: (a) T‐cell panel: CD3‐V450, CD4‐PerCP‐Cy5.5, CD45RA‐PE‐Cy7, CCR7‐PE, CD38‐APC, CD8‐APC‐H7, HLA‐DR‐V500 (BD Biosciences), CXCR3‐AF488, CCR6‐BV605, and CD45‐AF700 (BioLegend); (b) Treg panel: CD4‐PerCP‐Cy5.5, CD25‐PE, CCR4‐PE‐Cy7, CD127‐AF647, CD45RO‐APC‐H7, CD3‐V450, HLA‐DR‐V500 (BD Biosciences), and CD45‐AF700 (BioLegend); (c) B‐cell panel: CD24‐FITC, CD19‐PerCP‐Cy5.5, CD38‐APC, CD20‐APC‐H7, CD3‐V500 (BD Biosciences), IgD‐PE‐Cy7, CD27‐BV421, and CD45‐AF700 (BioLegend); and (d) DC/monocyte/NK panel: CD3 + CD19‐APC‐H7, CD56‐PE, CD16‐APC, CD14‐V450, CD123‐PerCP‐Cy5.5, CD11c‐PE‐Cy7, HLA‐DR‐V500 (BD Biosciences), and slan‐FITC (Miltenyi Biotec).

##### Quantification of absolute cell numbers

For quantification of absolute lymphocyte and peripheral blood mononuclear cell (PBMC) counts, 25 µL of peripheral blood samples was incubated with anti‐CD45‐PerCP (BD Biosciences) for 20 min at room temperature and protected from the light. Erythrocytes were removed using 450 µL lysis buffer (BD FACS Lysing Solution; BD Biosciences). In order to quantify the absolute number of cells, Perfect‐Count Microspheres (Cytognos SL) were added to each sample in the same proportion of blood, 25 µL (ratio 1:1), and acquired on a LSR II Fortessa cytometer and analyzed using FACSDiva software (both from BD Biosciences). The number of total lymphocytes was expressed in cells/µL.

### 2.3 Statistical analysis

Statistical analyses were performed using GraphPad Prism version 6.00 for Windows. Parametric and nonparametric tests were used depending on the normality of the distribution of the variables. Differences between samples obtained at different time points were compared to baseline measurements using the repeated measures 1‐way ANOVA test with Dunnett's or Dunn's correction for multiple comparisons. To compare data from NEDA and ODA groups, Mann‐Whitney U or t tests were applied. Fisher's exact test was used to compare qualitative variables. Results were expressed as mean ± standard deviation (SD) values, unless otherwise stated. Differences were considered statistically significant when *P* < 0.05.

## 3 RESULTS

### 3.1 Patients

A total of 22 MS patients were enrolled in the study. To determine the effects of DMF related to an optimal response, 21 out of 22 patients were classified as NEDA (n = 15) or ODA (n = 6) patients. One patient was excluded from this stratification due to the lack of MRI at 12 months. No differences were found on baseline characteristics between groups, and, as expected, the ODA group showed a higher number of relapses (*P* = 0.0016) and an increased presence of new T2 lesions (*P* = 0.0025) after 12 months of follow‐up (Table 1).

### 3.2 Optimal response to DMF is related to a reduction of memory CD4^+^ T cells

As has been widely described, the analysis of whole blood samples revealed that DMF induced a progressive decrease in the absolute number of lymphocytes (*P* = 0.0005). This reduction was statistically significant after 6 months of treatment and remained after 12 months of follow‐up (Figure S1A and Table S2).

When the effect of DMF on T cells was investigated, a decrease in the percentage of CD3^+^ T cells was found (*P* < 0.0001) following just 3 months of treatment (Figure S1B). Further analyses revealed that the number of both CD8^+^ and CD4^+^ T cells was reduced although CD4^+^ lymphocytes were decreased in a lesser extent (Table S2). Consequently, the proportion of CD4^+^ T cells and the ratio of CD4^+^/CD8^+^ T lymphocytes were significantly increased at 6 and 12 months of DMF treatment (*P* < 0.0001 and *P* = 0.0001, respectively) (Figure S1C).

**Figure 1:**
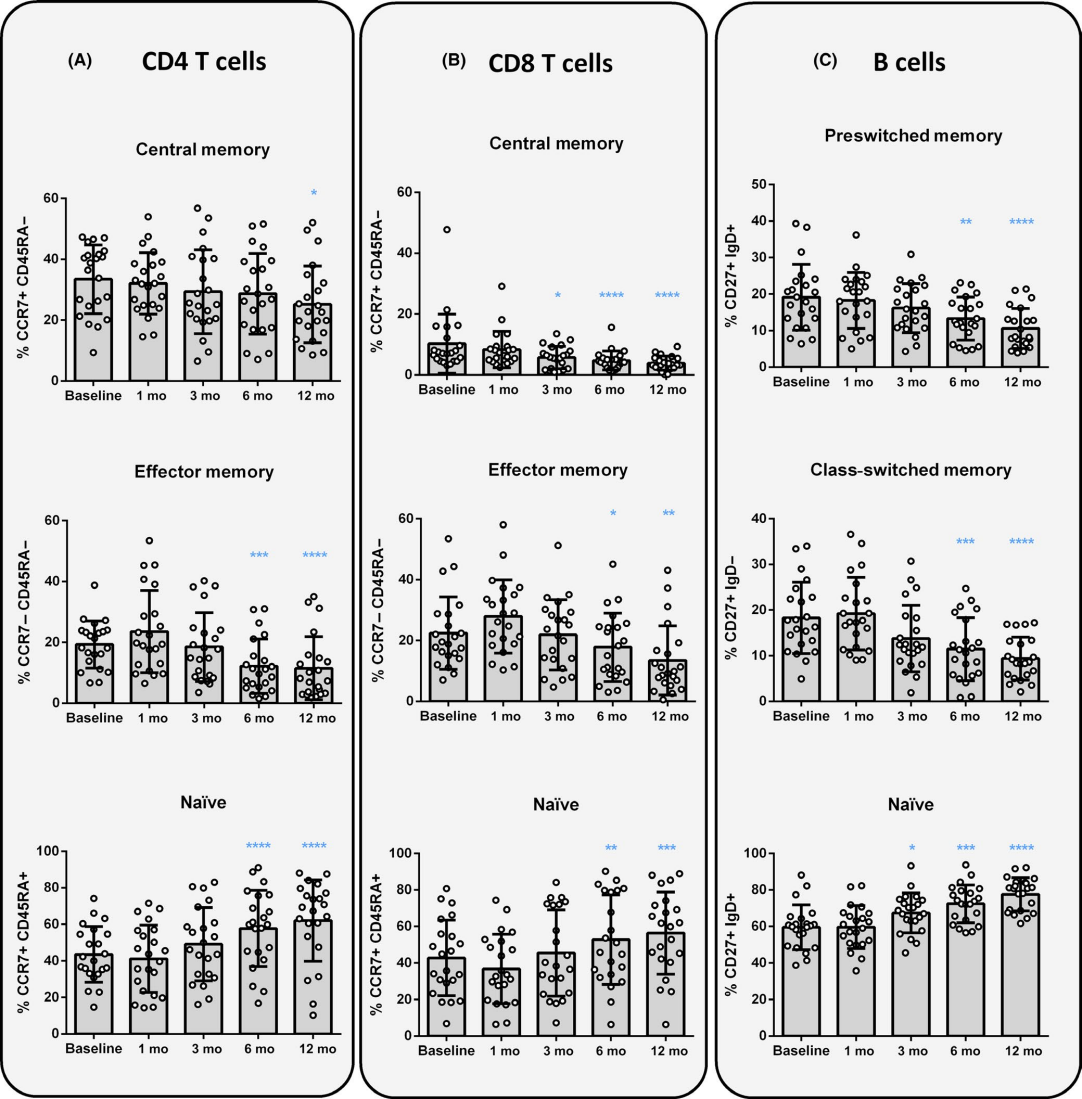
Changes induced by DMF treatment on T‐ and B‐lymphocyte subpopulations. Representation of percentages (%) of central memory (CCR7^+^ CD45RA^−^), effector memory (CCR7^−^ CD45RA^−^), and naïve (CCR7^+^ CD45RA^+^) subpopulations from CD4^+^ and CD8^+^ T lymphocytes (A and B, respectively); and % of preswitched memory (CD27^+^ IgD^+^), class‐switched (CD27^+^ IgD^−^) and (CD27^−^ IgD^+^) naïve CD19^+^ B lymphocytes (C), before the treatment (baseline) and after 1, 3, 6 and, 12 months of follow‐up. Data are expressed as mean SD. Each dot represents the value of an individual (n = 22). *P < 0.05, **P < 0.01, ***P < 0.001, ****P < 0.0001

**Figure 2:**
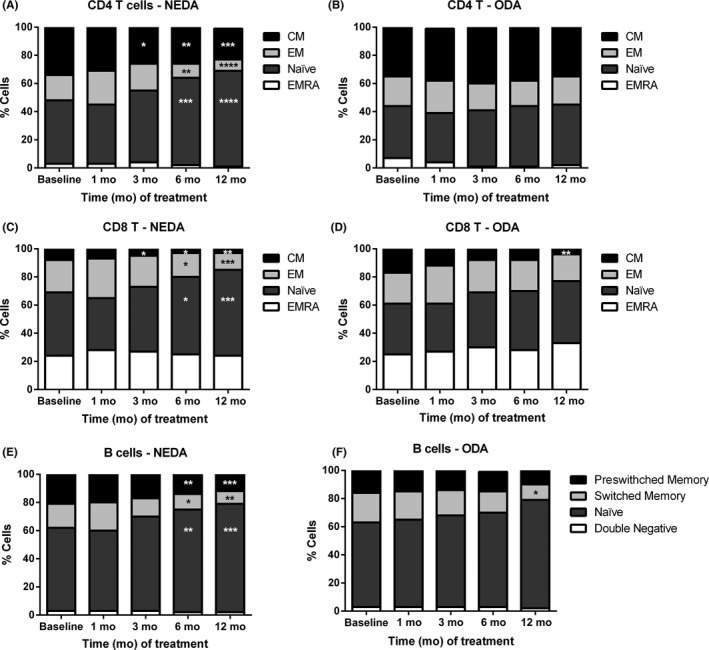
DMF specifically depleted memory T‐ and B‐lymphocyte subpopulations in  NEDA patients. Representation of percentages (%) of central memory (CM), effector memory (EM), naïve, and terminally differentiated RA (EMRA) CD4^+^ and CD8^+^ T lymphocytes from patients with no evidence of disease activity (NEDA) (A, C) and patients with ongoing disease activity (ODA) (B, D), before the treatment (Baseline) and after 1, 3, 6, and 12 months of follow‐up. In addition, % of preswitched memory, class‐switched, naïve, and double negative B lymphocytes from NEDA and ODA patients (E, F) was analyzed before and after treatment. Data are expressed as mean of each lymphocyte subpopulation of NEDA (n = 15) and ODA (n = 6) patients. *P < 0.05, **P < 0.01, ***P < 0.001, ****P < 0.0001

A deeper analysis on T‐lymphocyte subpopulations showed a progressive depletion of T central memory (CD4: *P* = 0.0106; CD8: *P* < 0.0001) and effector memory cells (CD4 and CD8: *P* < 0.0001), resulting in an increase of the relative percentage of naïve CD4^+^ and CD8^+^ lymphocytes (CD4 and CD8: *P* < 0.0001) (Figure 1A,B and Table S3). These changes were statistically significant at 6 and 12 months of treatment for effector memory CD4^+^ and CD8^+^ T cells. In the case of central memory CD8^+^ and CD4^+^ T lymphocytes, differences were found after 3 or 12 months of follow‐up, respectively. Interestingly, when patients were classified as NEDA or ODA, the effects caused by the DMF treatment on CD4^+^ and CD8^+^ T‐lymphocyte subpopulations were only observed in NEDA patients, but not in ODA patients, with the exception of CD8^+^ central memory cells, which were reduced in both groups of patients (Figure 2A‐D and Table S3). In addition, NEDA patients exhibited a significant reduction of both CD4^+^ and CD8^+^ central memory T lymphocytes after 3 months of treatment (*P* < 0.0001 and *P* = 0.0021, respectively) (Figure 2A,C and Table S3).

**Figure 3:**
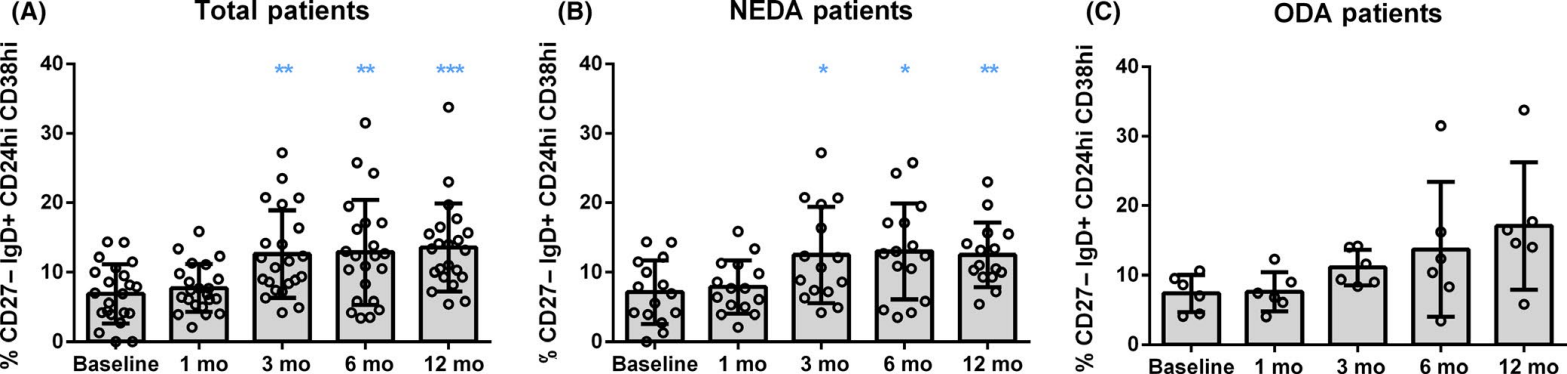
Transitional B cells increased in  NEDA patients following DMF treatment. Representation of percentages (%) of transitional B lymphocytes (CD27^−^ IgD^+^ CD24hi CD38hi) from the total of B cells (CD3^−^ CD19^+^ cells), before the treatment (Baseline) and after 1, 3, 6, and 12 months of follow‐up in total RRMS patients (n = 22) (A), and after been classified in NEDA (n = 15) (B) and ODA (n = 6) (C) groups. Data are expressed as mean SD. Each dot represents the value of an individual. *P < 0.05, **P < 0.01 and ***P < 0.001

The analysis of B‐cell subsets revealed comparable results to those found in T‐cell subpopulations (Tables S4 and S5). Although the total percentage of B cells remained stable (Figure S1C), DMF induced a specific depletion of memory B cells, both switched and preswitched class cells (*P* < 0.0001), thus increasing the percentage of naïve B cells (*P* < 0.0001) (Figure 1C). Moreover, transitional B cells were already found increased in both, percentage and absolute cell counts, after 3 months of treatment (*P* < 0.0001) (Figure 3A). No changes in double negative (CD27^−^ IgD^−^) B cells, and plasma cells were observed on DMF‐treated patients (Tables S4 and S5).

The analysis of NEDA and ODA patients showed that B cells from NEDA and ODA patients were affected in a similar way, although the group of ODA patients did not reach statistical differences in most of the time points compared to baseline (probably due to the low number of samples of the ODA group) (Figure 2E,F): (a) decrease of preswitched and switched class memory B cells (NEDA: *P* < 0.0001 and *P* = 0.0005 vs ODA: *P* = 0.2148 and *P* = 0.0049, respectively); (b) increase of naïve B cells (NEDA: *P* < 0.0001 vs ODA: *P* = 0.0415); and (c) increase of transitional B cells (NEDA: *P* = 0.0006 vs ODA *P* = 0.0791) (Figure 3B,C and Table S5).

### 3.3 DMF reduces Th1‐like Th17 lymphocytes in NEDA patients

**Figure 4:**
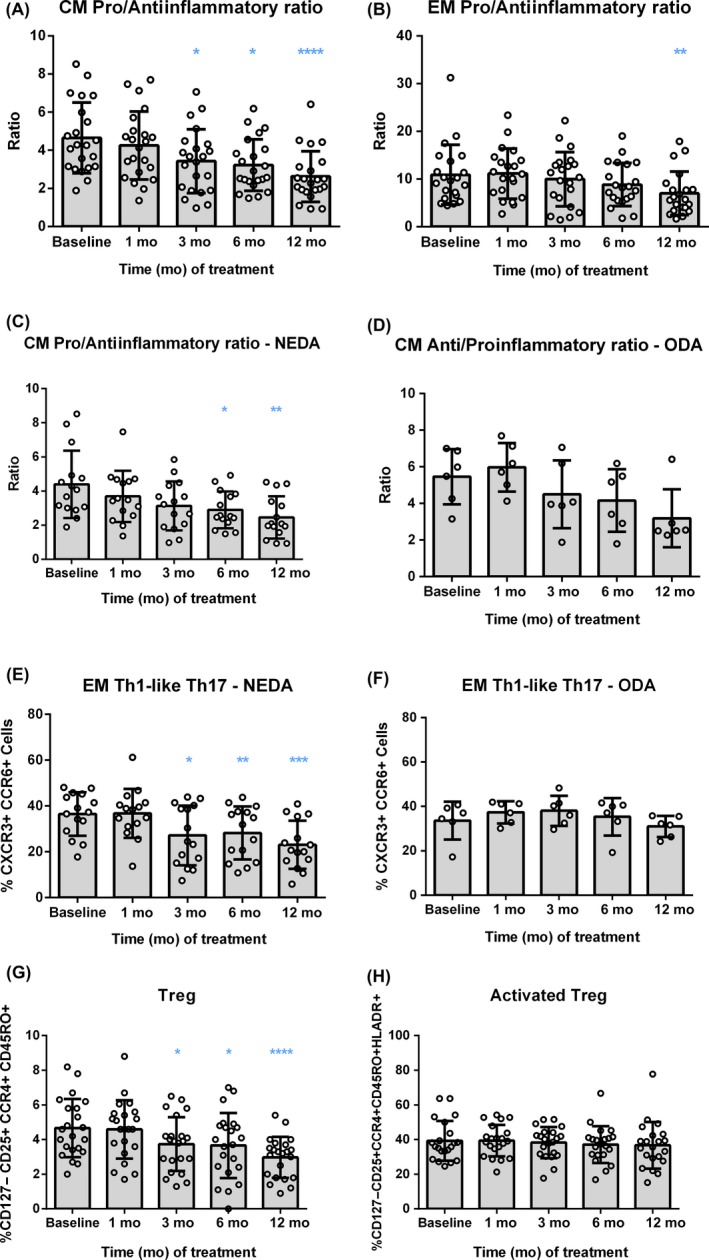
Selective depletion of the pro‐inflammatory Th1‐like Th17 cells in  NEDA patients after 3 months of DMF treatment. Representation of the pro‐/antiinflammatory ratio in central memory (CM) and effector memory (EM) CD4^+^ T cells for total RRMS patients (n = 22) (A, B) as well as for NEDA and ODA patients (n = 15 and n = 6, respectively) (C, D), before the treatment (Baseline) and after 1, 3, 6, and 12 months of follow‐up. For ratio calculations, the % of Th2 cells was considered as antiinflammatory and the sum of the % of Th1, Th17, and Th1‐like Th17 was used as pro‐inflammatory values. The % of Th1‐like Th17 (CXCR3^+^ CCR6^+^) T lymphocytes was analyzed on NEDA (E) and ODA (F) patients. In addition, the % of regulatory T cells (Treg, CD127^−^ CD25 +CCR4^+^ CD45RO^+^) (G) and activated Treg (HLA‐DR^+^ Treg) (H) were determined. Data are expressed as mean SD. Each dot represents the value of an individual. *P < 0.05, **P < 0.01, ***P < 0.001, ****P < 0.0001

When the pro‐ and antiinflammatory effects of DMF were evaluated (Figure S2) by analyzing whole blood samples, a reduction of the Th1‐like Th17 population was found after 6 and/or 12 months of treatment in central memory and effector memory cells, respectively (*P* < 0.0001 in both subsets, Figure S3). Importantly, an increase in the percentage of central and effector memory Th2 lymphocytes was also observed (*P* < 0.0001 and *P* = 0.0065, respectively). This antiinflammatory effect was significantly relevant after 3 months of treatment for central memory Th2 cells and after 12 months in the case of effector memory Th2 cells (Table S3). Surprisingly, the analysis exhibited an increase of effector memory Th17 cells after 6 and 12 months of follow‐up (*P* < 0.0001). However, when the global pro‐ and antiinflammatory balance was compared in central and effector memory cells, a progressive decrease of the pro‐inflammatory cells was evidenced (*P* < 0.0001 and *P* = 0.0065, respectively) after 3 months of DMF treatment on central memory cells and after 12 months on effector memory cells (Figure 4A,B, respectively) (Tables S2 and S3).

The stratification of patients according to their clinical and radiological activity revealed similar effects of DMF on the different pro‐ and antiinflammatory lymphocytes subpopulations, being only statistically significant in NEDA patients (Figure 4C,D). It is worth noting that a reduction in the percentage of effector memory Th1‐like Th17 cells was detected in NEDA patients from month 3 to month 12 of follow‐up, but no differences were found in the ODA group (Figure 4E,F, and Table S3). Interestingly, differences in the percentage of Th1‐like Th17 effector memory cells between NEDA and ODA patients were statistically significant after 3 and 6 months of treatment (*P* = 0.0202 and *P* = 0.0373, respectively).

On the other hand, the analysis of memory Treg in the whole group of MS patients showed a progressive reduction of this population, being statistically significant after 3 months of DMF treatment (*P* = 0.0001; Figure 4G). However, the percentage of activated memory Treg remained stable during the whole observation period (Figure 4H). Results obtained considering NEDA and ODA patients were similar for both memory Treg and activated Treg subpopulations (Tables S2 and S3).

### 3.4 DMF shifts the balance between cytotoxic and regulatory NK cells

**Figure 5:**
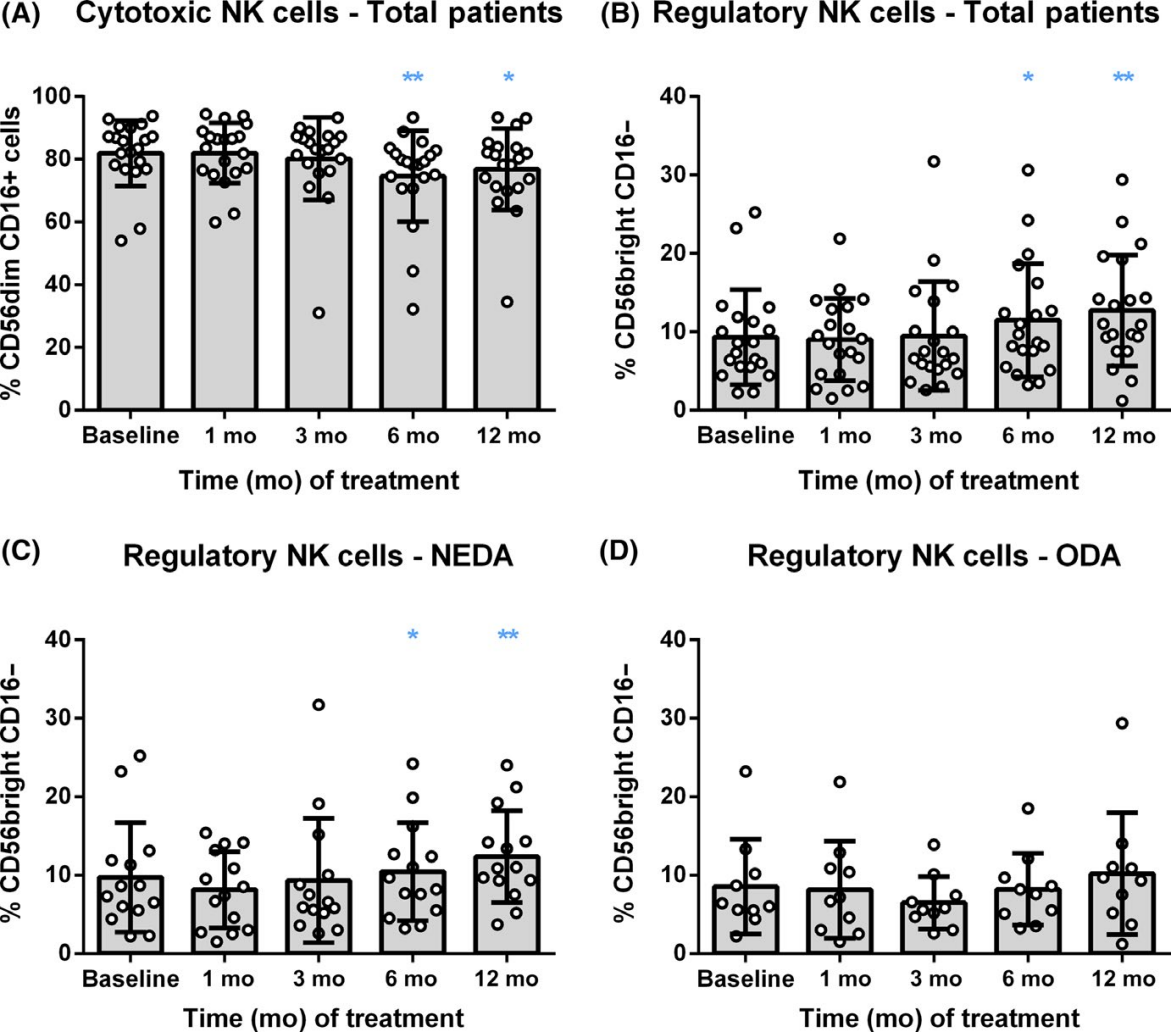
DMF shifted the balance from cytotoxic to regulatory NK cells. Representation of percentage (%) of cytotoxic (CD56dim CD16^+^) and regulatory (CD56bright CD16‐) natural killer (NK) cells (A and B, respectively), before the treatment (Baseline) and after 1, 3, 6, and 12 months of follow‐up in total RRMS patients (n = 22); and regulatory NK cells in NEDA (n = 15) (C) and ODA (n = 6) (D) patients. Data are expressed as mean SD. Each dot represents the value of an individual. *P < 0.05 and **P < 0.01

The evaluation of percentages and absolute cell counts of monocytes, natural killer (NK) cells, and dendritic cells (DC) did not show any alteration of these subpopulations caused by DMF treatment. However, after 6 months of follow‐up, the percentage of circulating cytotoxic CD56^dim^ CD16^+^ NK cells was reduced (*P* = 0.0032), while the proportion of immunoregulatory CD56^bright^ NK cells increased (*P* = 0.0003) (Figure 5A,B). The analysis in NEDA and ODA patients revealed that the ODA group did not reach a statistical increase of regulatory NK cells (NEDA, *P* = 0.0008; ODA, *P* = 0.4152) (Figure 5C,D; and Tables S4 and S5).

## 4 DISCUSSION

During the last years, the multiple treatment options available for MS patients have driven the attention of physicians and researchers on identifying the most suitable DMT depending not only on the clinical and immunological characteristics of the patient, but also on the specific mechanism of action of each therapy and its potential risks. To this end, cross‐sectional studies have contributed notably to elucidate the most relevant effects of different DMT such as DMF or fingolimod. However, due to the interindividual heterogenicity, some less obvious changes could remain undetectable. In contrast, longitudinal studies are limited by the final number of patients completing the follow‐up period, but results obtained avoid interindividual variability and are useful to monitor changes induced by the treatment over time. With this purpose, our group has been collaborating in an international consortium to establish standardized multiparametric cytometric protocols to evaluate changes in leukocyte subpopulations of patients with autoimmune diseases such as type I diabetes, rheumatoid arthritis, or MS^15^, ^16^ Importantly, these cytometric panels are performed on fresh whole blood samples, providing an easy, fast, and direct approach to obtain a readout of circulating leukocytes of patients able to be translated into clinical practice.

Although etiology of MS still remains unknown, it is considered an immune‐mediated disease in which myelin‐reactive CD4^+^ T lymphocytes trigger a complex immune response that leads to inflammation, myelin destruction, and axonal loss.^17^, ^18^, ^19^ The main players initiating the autoimmune attack are autoreactive CD4^+^ Th1 and Th17 cells, which produce pro‐inflammatory cytokines, such as IFN‐γ and IL‐17, respectively. These cells are able to cross the blood‐brain barrier and reach the CNS and recognize myelin antigens, secreting pro‐inflammatory mediators and activating other immune cells, such as B cells and cytotoxic CD8^+^ T cells.^20^ Consequently, different pharmacological strategies have been developed to abrogate or block these immune cascades.

In this study, we prospectively evaluated changes induced by DMF treatment performing a direct whole blood cytometric analysis. This approach provided a deep analysis of the adaptive and innate immune response, being able to reproduce most of the DMF effects reported during last years.

As described, DMF reduced the absolute number of circulating lymphocytes, affecting more extensively CD8^+^ cytotoxic T cells^8^, ^21^ and therefore increasing the CD4^+^/CD8^+^ T‐cell ratio.^9^, ^22^ Further analyses evidenced the characteristic phenotypic alterations produced by DMF treatment on CD4^+^ and CD8^+^ T‐cell subpopulations, a selective reduction of central and effector memory T cells, and an expansion of naïve T cells. This effect of DMF is considered as one of the most important effects reducing MS activity on patients, since memory cells are responsible to induce a rapid and efficient response against a previously recognized antigen. While effector memory T cells rapidly migrate to the foci of inflammation to orchestrate a rapid immune response, central memory T cells home to lymph nodes, proliferate following antigen restimulation, and then differentiate into effector memory T cells. Therefore, the depletion of memory cells could be beneficial to reduce disease activity. In this regard, we observed that only NEDA patients were exhibiting a significant decrease of CD4^+^ central and effector memory subpopulations, as well as CD8^+^ effector memory T cells.

Interestingly, the expression analysis of CXCR3 and CCR6, surrogate markers to define Th1 (CXCR3^+^ CCR6^−^), Th17 (CXCR3^−^ CCR6^+^), Th1‐like Th17 (CXCR3^+^ CCR6^+^), and Th2 (CXCR3^−^ CCR6^−^) CD4^+^ lymphocyte subpopulations revealed an expansion of antiinflammatory Th2 cells (both central memory and effector memory), together with a marked decrease of central memory and effector memory Th1‐like Th17 cells. These Th1‐like Th17 lymphocytes, also described as Th1/Th17, are Th17 cells with Th1‐like features, producing both IFN‐γ and Th17 pro‐inflammatory cytokines.^23^, ^24^ In a recent publication, Langelaar et al^25^ described that effector memory Th1‐like Th17 express the α4β1 integrin, very late antigen‐4 (VLA‐4), and therefore, their activation promotes their migration into the CNS, inducing neuroinflammation. In our study, we showed for the first time that DMF exerts an antiinflammatory effect by increasing Th2 cells and decreasing Th1‐like Th17 cells. Surprisingly, a reduction in the proportion of circulating memory Treg was found. Although not addressed in this work, it would be interesting to analyze the expression of CXCR3 and CCR6 in the Treg population to know whether this reduction is related to a decrease of pro‐inflammatory Th1 and/or Th17 Treg.^26^, ^27^ Regarding discrepancies with other studies reporting a reduction on Th1 and Th17 cells and no alteration or even expansion of Treg,^8^, ^9^, ^12^ they might be caused by the use in those studies of isolated, cryopreserved, and stimulated PBMC (since it has been reported that these procedures can modify the expression of chemokine receptors).^28^, ^29^, ^30^ We consider that the direct analysis of whole blood leukocytes subpopulations can reflect better the effects caused by DMF treatment, avoiding variability caused by cell manipulation. Strikingly, our results showed a reduction of effector memory Th1‐like Th17 cells only in NEDA patients, already after 3 months of treatment, meaning that this population could be a candidate biomarker for the early identification of optimal DMF responders.

On the other hand, we also observed a selective decrease on circulating memory B cells (both switched and preswitched), in parallel with an increase of naïve B lymphocytes, in accordance with previous publications.^10^, ^22^, ^31^ B cells can produce pro‐inflammatory cytokines, and treatments depleting the B‐cell compartment such as rituximab (anti‐CD20 monoclonal antibody) have demonstrated beneficial effects on MS patients. However, DMF not only mediated a depletion of mature or differentiated B cells, but also an increase of transitional B cells, a population of cells enriched in regulatory IL‐10 producing B cells. It was not addressed whether this increase was due to an increase of transitional B cells or to a suppression of their differentiation to naïve mature B cells. This finding is in accordance with previous studies also showing an increase of transitional B cells following 12 months of treatment,^22^, ^31^, ^32^ although our work is the first reporting this effect after only 3 months of DMF therapy.

Among monocytes, DC, and NK cells, only NK‐cell subpopulations were affected by DMF treatment. Specifically, this treatment reduced cytotoxic CD56^dim^ CD16^+^ NK cells and increased the percentage of circulating CD56^bright^ CD16^−^ NK cells after 6 months of follow‐up. NK cells are an important component of innate immunity that can be divided in 2 main subsets, CD56^dim^ CD16^+^ and CD56^bright^ CD16^−^ NK cells. The most frequent NK‐cell population in peripheral blood are the CD56^dim^CD16^+^ cells, which have perforin‐mediated cytolytic activity. In contrast, CD56^bright^CD16^−^ NK cells are predominant in secondary lymphoid tissues and have high proliferative activity and cytokine production, both pro‐ and antiinflammatory, depending on the context. It is worth noting that clinical efficacy of daclizumab in MS patients has been correlated with an expansion of this CD56^bright^ NK cells.^33^ Consequently, another mechanism mediating DMF efficacy could be related to the reduction of cytotoxic NK cells as well as the expansion of regulatory CD56^bright^ NK cells induced by the treatment. In this regard, our data indicate that only the expansion of CD56^bright^ CD16^−^ NK cells was related to the optimal response of DMF, since a statistically significant change was not reached in ODA patients. These results are in accordance with the study of Medina et al,^12^ which found a negative correlation between CD56^bright^ NK cells and various cytokine‐producing T cells, postulating that this CD56^bright^ NK cells could inhibit pathogenic effector lymphocytes. In this regard, our study has revealed a reduction of Th1‐like Th17 effector memory lymphocytes, a population of IFN‐ϒ and IL‐17 secreting cells that might be downregulated by the action of CD56^bright^ NK cells. Moreover, among Th1‐like Th17 cells, Th17.1 lymphocytes (CCR6^+^CXCR3^+^CCR4^−^) are a subpopulation of lymphocytes with superior capacity to reach the CNS. It was found selectively accumulated in peripheral blood of natalizumab‐treated MS patients who did not experience clinical relapses.^25^Further studies are needed to investigate whether the effect of DMF increasing CD56^bright^ NK cells mediates the reduction of Th17.1 cells, promoting, consequently, the reduction of neuroinflammation in NEDA patients.

In summary, we have shown that whole blood multiparametric flow cytometry analysis is a useful tool to perform an exhaustive immune monitoring of MS patients under DMF treatment. Our longitudinal study demonstrated that DMF mediates its beneficial effect by depleting memory T and B cells, as well as cytotoxic NK cells, while increasing the prevalence of transitional B cells and CD56^bright^ NK cells, shifting the balance from a pro‐ to an antiinflammatory profile.

Furthermore, we have identified effector memory Th1‐like Th17 lymphocytes as a potential biomarker for the early identification of DMF responder patients. However, further studies with larger numbers and independent cohorts of MS patients are necessary to confirm these results.

## CONFLICT OF INTEREST

The authors declare no conflict of interest.
