# Supplementary figures and images for: Optimal response to dimethyl fumarate is mediated by a reduction of Th1‐like Th17 cells after 3 months of treatment
Source: CNS Neurosci Ther. 2019 May 7;25(9):995–1005. doi: 10.1111/cns.13142 (PMC6698982; doi:10.1111/cns.13142)

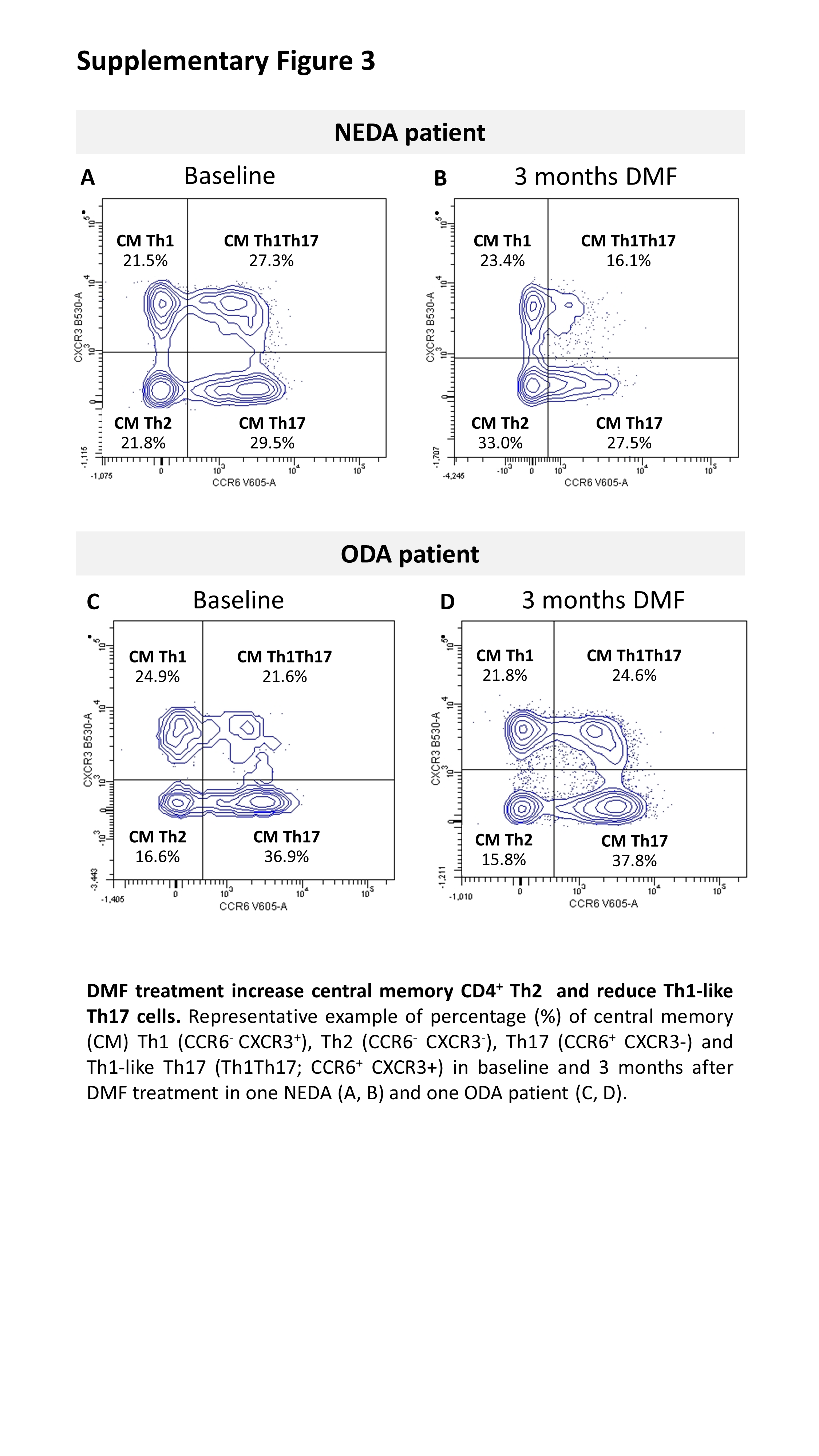

Supplement: Supplementary file 3 [file CNS-25-995-s003.tif]
