# Supplementary material for: Optimal response to dimethyl fumarate is mediated by a reduction of Th1‐like Th17 cells after 3 months of treatment
Source: CNS Neurosci Ther. 2019 May 7;25(9):995–1005. doi: 10.1111/cns.13142 (PMC6698982; doi:10.1111/cns.13142)
Supplement: Supplementary file 4 [file CNS-25-995-s004.docx]

**Supplementary Table 1. Phenotype of leukocyte subpopulations**

| **Leukocyte subpopulation** | **Phenotype** |
| --- | --- |
| **T cells** | **CD45^+^ CD3^+^** |
| **CD4^+^ T cells** | CD45^+^ CD3^+^ **CD4^+^** |
| CD4^+^ Naïve T cells | CD45^+^ CD3^+^ CD4^+^ **CCR7^+^ CD45RA^+^** |
| CD4^+^ Central Memory T cells | CD45^+^ CD3^+^ CD4^+^ **CCR7^+^ CD45RA^-^** |
| Th1 | CD45^+^ CD3^+^ CD4^+^ CCR7^+^ CD45RA^-^ **CCR6^-^ CXCR3^+^** |
| Th2 | CD45^+^ CD3^+^ CD4^+^ CCR7^+^ CD45RA^-^ **CCR6^-^ CXCR3^-^** |
| Th17 | CD45^+^ CD3^+^ CD4^+^ CCR7^+^ CD45RA^-^ **CCR6^+^ CXCR3^-^** |
| Th17.1 | CD45^+^ CD3^+^ CD4^+^ CCR7^+^ CD45RA^-^ **CCR6^+^ CXCR3^+^** |
| CD4^+^ Effector Memory T cells | CD45^+^ CD3^+^ CD4^+^ **CCR7^-^ CD45RA^-^** |
| Th1 | CD45^+^ CD3^+^ CD4^+^ CCR7^-^ CD45RA^-^ **CCR6^-^ CXCR3^+^** |
| Th2 | CD45^+^ CD3^+^ CD4^+^ CCR7^-^ CD45RA^-^ **CCR6^-^ CXCR3^-^** |
| Th17 | CD45^+^ CD3^+^ CD4^+^ CCR7^-^ CD45RA^-^ **CCR6^+^ CXCR3^-^** |
| Th1-like Th17 | CD45^+^ CD3^+^ CD4^+^ CCR7^-^ CD45RA^-^ **CCR6^+^ CXCR3^+^** |
| Terminally differentiated effector memory CD4^+^ T cells | CD45^+^ CD3^+^ CD4^+^ **CCR7^-^ CD45RA^+^** |
| **Treg** | CD45^+^ CD3^+^ CD4^+^ **CD127^-^ CD25^+^ CCR4^+^** |
| Memory Treg | CD45^+^ CD3^+^ CD4^+^ CD127^-^ CD25^+^ CCR4^+^ **CD45RO^+^** |
| Activated memory Treg | CD45^+^ CD3^+^ CD4^+^ CD127^-^ CD25^+^ CCR4^+^ CD45RO^+^ **HLA-DR^+^** |
| **CD8^+^ T cells** | CD45^+^ CD3^+^ **CD8^+^** |
| CD8^+^ Naïve T cells | CD45^+^ CD3^+^ CD8^+^ **CCR7^+^ CD45RA^+^** |
| CD8^+^ Central Memory T cells | CD45^+^ CD3^+^ CD8^+^ **CCR7^+^ CD45RA^-^** |
| CD8^+^ Effector Memory T cells | CD45^+^ CD3^+^ CD8^+^ **CCR7^-^ CD45RA^-^** |
| Terminally differentiated effector memory CD8^+^ T cells | CD45^+^ CD3^+^ CD8^+^ **CCR7^-^ CD45RA^+^** |
| **B cells** | **CD45^+^ CD3^-^ CD19^+^** |
| Naïve B cells | CD45^+^ CD3^-^ CD19^+^ **CD27^-^ IgD^+^** |
| Transitional B cells | CD45^+^ CD3^-^ CD19^+^ CD27^-^ IgD^+^ **CD24hi CD38hi** |
| Pre-switched Memory B cells | CD45^+^ CD3^-^ CD19^+^ **CD27^+^ IgD^+^** |
| Switched Memory B cells | CD45^+^ CD3^-^ CD19^+^ **CD27^+^ IgD^-^** |
| Plasma cells | CD45^+^ CD3^-^ CD19^+^ CD27^+^ IgD^-^ **CD38hi** |
| Double negative (CD27^-^ IgD^-^) B cells | CD45^+^ CD3^-^ CD19^+^ **CD27^-^ IgD^-^** |
| **Natural Killer (NK) cells** | **CD45^+^ CD3^-^ CD19^-^ CD56^+^** |
| CD56^bright^ CD16^-^ NK cells | CD45^+^ CD3^-^ CD19^-^ **CD56^bright^ CD16^-^** |
| CD56dim CD16^+^ NK cells | CD45^+^ CD3^-^ CD19^-^ **CD56dim CD16^+^** |
| **Monocytes** | **CD45^+^ CD3^-^ CD19^-^ CD14^+^** |
| Classical Monocytes | CD45^+^ CD3^-^ CD19^-^ **CD14^+^ CD16^+^** |
| Nonclassical Monocytes | CD45^+^ CD3^-^ CD19^-^ **CD14low CD16^+^** |
| **Dendritic Cells (DC)** | **CD45^+^ CD3^-^ CD19^-^ CD56^-^ CD14^-^ HLA-DR^+^** |
| Myeloid DC | CD45^+^ CD3^-^ CD19^-^ CD56^-^ CD14^-^ HLA-DR^+^ **CD11c^+^** |
| Plasmacytoid DC | CD45^+^ CD3^-^ CD19^-^ CD56^-^ CD14^-^ HLA-DR^+^ **CD123^+^** |
