# Supplementary material for: Optimal response to dimethyl fumarate is mediated by a reduction of Th1‐like Th17 cells after 3 months of treatment
Source: CNS Neurosci Ther. 2019 May 7;25(9):995–1005. doi: 10.1111/cns.13142 (PMC6698982; doi:10.1111/cns.13142)
Supplement: Supplementary file 5 [file CNS-25-995-s005.docx]

**Supplementary table 2**. Absolute counts of T cell subpopulations in RRMS patients under dimethyl fumarate treatment during 12 month follow-up.

|  | **Baseline** | | | **+1month** | | | **+3month** | | | **+6month** | | | **+12month** | | |
| --- | --- | --- | --- | --- | --- | --- | --- | --- | --- | --- | --- | --- | --- | --- | --- |
|  | **(cells/µl)** | | | **(cells/µl)** | | | **(cells/µl)** | | | **(cells/µl)** | | | **(cells/µl)** | | |
| **Total PBMC** ^Ϯ^ | 2023 | ± | 618 | 1922 | ± | 653 | 1850 | ± | 809 | 1493 | ± | 563 | 1593 | ± | 889 |
| **Total lymphocytes** | 1542 | ± | 570 | 1419 | ± | 522 | 1393 | ± | 687 | 1098 | ± | 450 | 1102 | ± | 646 |
| **T cell subsets** |  |  |  |  |  |  |  |  |  |  |  |  |  |  |  |
| **CD3 lymphocytes** | 1212 | ± | 32 | 1080 | ± | 35 | 1041 | ± | 37 | 821 | ± | 24 | 791 | ± | 52 |
| **CD4+ T cell subsets** |  |  |  |  |  |  |  |  |  |  |  |  |  |  |  |
| **CD4 lymphocytes** |  | ± | 4 | 635 | ± | 3 | 640 | ± | 3 | 530 | ± | 2 | 524 | ± | 5 |
| **CD4+ Naïve T cells** | 305 | ± | 1 | 261 | ± | 1 | 315 | ± | 1 | 306 | ± | 0 | 325 | ± | 1 |
| **CD4+ Central memory T cells** | 234 | ± | 0 | 204 | ± | 0 | 188 | ± | 0 | 152 | ± | 0 | 132 | ± | 1 |
| **Th1 Central memory** | 67 | ± | 0 | 53 | ± | 0 | 44 | ± | 0 | 36 | ± | 0 | 34 | ± | 0 |
| **Th2 Central memory** | 46 | ± | 0 | 43 | ± | 0 | 49 | ± | 0 | 39 | ± | 0 | 41 | ± | 0 |
| **Th17 Central memory** | 59 | ± | 0 | 55 | ± | 0 | 54 | ± | 0 | 46 | ± | 0 | 36 | ± | 0 |
| **Th1/Th17 Central memory** | 62 | ± | 0 | 52 | ± | 0 | 41 | ± | 0 | 31 | ± | 0 | 21 | ± | 0 |
| **CD4+ Effector memory T cell** | 135 | ± | 0 | 149 | ± | 0 | 118 | ± | 0 | 65 | ± | 0 | 60 | ± | 1 |
| **Th1 Effector memory** | 52 | ± | 0 | 55 | ± | 0 | 40 | ± | 0 | 23 | ± | 0 | 21 | ± | 0 |
| **Th2 Effector memory** | 14 | ± | 0 | 16 | ± | 0 | 15 | ± | 0 | 8 | ± | 0 | 10 | ± | 0 |
| **Th17 Effector memory** | 21 | ± | 0 | 25 | ± | 0 | 28 | ± | 0 | 14 | ± | 0 | 14 | ± | 0 |
| **Th1/Th17 Effector memory** | 48 | ± | 0 | 54 | ± | 0 | 35 | ± | 0 | 20 | ± | 0 | 15 | ± | 0 |
| **CD4+ T_EMRA_** | 27 | ± | 0 | 21 | ± | 0 | 36 | ± | 0 | 8 | ± | 0 | 7 | ± | 0 |
| **T regulatory subsets** |  |  |  |  |  |  |  |  |  |  |  |  |  |  |  |
| **Memory Treg** | 60 | ± | 0 | 54 | ± | 0 | 52 | ± | 0 | 42 | ± | 0 | 41 | ± | 0 |
| **Activated memory Treg** | 24 | ± | 0 | 21 | ± | 0 | 20 | ± | 0 | 15 | ± | 0 | 15 | ± | 0 |
| **CD8+ T cell subsets** |  |  |  |  |  |  |  |  |  |  |  |  |  |  |  |
| **CD8 lymphocytes** | 441 | ± | 3 | 386 | ± | 3 | 349 | ± | 3 | 261 | ± | 2 | 237 | ± | 4 |
| **CD8+ Naïve T cell** | 188 | ± | 1 | 142 | ± | 1 | 158 | ± | 1 | 138 | ± | 0 | 134 | ± | 1 |
| **CD8+ Central memory T cell** | 45 | ± | 0 | 32 | ± | 0 | 20 | ± | 0 | 12 | ± | 0 | 9 | ± | 0 |
| **CD8+ Effector memory T cell** | 99 | ± | 0 | 108 | ± | 0 | 76 | ± | 0 | 46 | ± | 0 | 32 | ± | 0 |
| **CD8+ T_EMRA_** | 108 | ± | 0 | 104 | ± | 0 | 94 | ± | 0 | 65 | ± | 0 | 63 | ± | 1 |

^Ϯ^ PBMC= Peripheral blood mononuclear cells.

Data shown in blue indicate statistically significant differences compared to baseline levels (p<0.05).

Th1/Th17= Th1-like Th17 cells
