# Supplementary material for: Optimal response to dimethyl fumarate is mediated by a reduction of Th1‐like Th17 cells after 3 months of treatment
Source: CNS Neurosci Ther. 2019 May 7;25(9):995–1005. doi: 10.1111/cns.13142 (PMC6698982; doi:10.1111/cns.13142)
Supplement: Supplementary file 6 [file CNS-25-995-s006.doc]

**Supplementary table 3.** Percentages of T lymphocyte subpopulations in total RRMS patients and groups of NEDA and ODA patients under dimethyl fumarate treatment during 12-months follow-up.

|  | **Baseline** | | | **+1month** | | | **+3month** | | | **+6month** | | | **+12month** | | |
| --- | --- | --- | --- | --- | --- | --- | --- | --- | --- | --- | --- | --- | --- | --- | --- |
|  | **(%)** | | | **(%)** | | | **(%)** | | | **(%)** | | | **(%)** | | |
| **T cell subsets** |  |  |  |  |  |  |  |  |  |  |  |  |  |  |  |
| **CD3 lymphocytes** | 78.6 | ± | 5.53 | 76.1 | ± | 6.74 | 74.6 | ± | 5.45 | 74.8 | ± | 5.29 | 71.8 | ± | 8.02 |
| **CD4+ T cell subsets** |  |  |  |  |  |  |  |  |  |  |  |  |  |  |  |
| **CD4 lymphocytes** | 57.8 | ± | 13.5 | 58.8 | ± | 9.93 | 61.5 | ± | 9.13 | 64.5 | ± | 9.23 | 66.2 | ± | 10.3 |
| **CD4+ Naïve T cells** | 43.5 | ± | 15.3 | 41.2 | ± | 18.4 | 49.1 | ± | 20.1 | 57.7 | ± | 20.9 | 62.1 | ± | 22.3 |
| **NEDA** | 45.2 | ± | 15.6 | 41.5 | ± | 16.9 | 50.8 | ± | 17.2 | 61.7 | ± | 17.3 | 68.5 | ± | 16.7 |
| **ODA** | 37.5 | ± | 15.0 | 35.2 | ± | 19.9 | 39.8 | ± | 23.7 | 43.3 | ± | 23.8 | 43.2 | ± | 26.5 |
| **CD4+ Central memory T cells** | 33.4 | ± | 11.3 | 32.1 | ± | 10.1 | 29.3 | ± | 13.8 | 28.7 | ± | 13.3 | 25.2 | ± | 12.6 |
| **NEDA** | 33.9 | ± | 9.99 | 31.1 | ± | 8.23 | 26.4 | ± | 11.2 | 26.4 | ± | 10.9 | 22.1 | ± | 9.52 |
| **ODA** | 34.5 | ± | 14.8 | 37.5 | ± | 11.9 | 40.0 | ± | 14.1 | 37.6 | ± | 14.9 | 35.6 | ± | 14.3 |
| **Th1 Central memory** | 28.8 | ± | 10.7 | 25.8 | ± | 7.18 | 23.3 | ± | 5.97 | 23.6 | ± | 5.82 | 26.1 | ± | 5.70 |
| **NEDA** | 26.5 | ± | 5.71 | 24.3 | ± | 5.20 | 22.9 | ± | 5.76 | 23.7 | ± | 5.69 | 25.8 | ± | 5.33 |
| **ODA** | 34.4 | ± | 18.3 | 28.4 | ± | 10.9 | 23.0 | ± | 6.58 | 22.9 | ± | 7.07 | 25.3 | ± | 6.17 |
| **Th2 Central memory** | 19.6 | ± | 6.25 | 21.4 | ± | 7.80 | 26.2 | ± | 10.7 | 26.0 | ± | 7.79 | 30.9 | ± | 10.2 |
| **NEDA** | 20.7 | ± | 6.73 | 23.5 | ± | 7.79 | 27.5 | ± | 10.8 | 27.5 | ± | 7.43 | 32.5 | ± | 11.3 |
| **ODA** | 16.3 | ± | 4.48 | 14.8 | ± | 2.94 | 20.2 | ± | 7.89 | 21.6 | ± | 8.28 | 25.8 | ± | 6.26 |
| **Th17 Central memory** | 25.3 | ± | 7.35 | 27.3 | ± | 5.87 | 28.9 | ± | 7.62 | 30.1 | ± | 6.24 | 27.2 | ± | 6.49 |
| **NEDA** | 16.9 | ± | 6.36 | 17.0 | ± | 6.74 | 26.5 | ± | 22.2 | 22.3 | ± | 10.2 | 24.2 | ± | 8.37 |
| **ODA** | 13.2 | ± | 7.14 | 14.7 | ± | 6.11 | 17.8 | ± | 6.35 | 21.0 | ± | 8.76 | 23.4 | ± | 9.79 |
| **Th1/Th17 Central memory** | 26.4 | ± | 8.53 | 25.6 | ± | 7.71 | 21.7 | ± | 8.68 | 20.4 | ± | 8.77 | 15.9 | ± | 7.50 |
| **NEDA** | 26.0 | ± | 9.46 | 24.5 | ± | 8.20 | 20.4 | ± | 8.43 | 18.8 | ± | 8.91 | 15.0 | ± | 8.00 |
| **ODA** | 33.6 | ± | 8.52 | 37.3 | ± | 4.95 | 38.0 | ± | 6.74 | 35.9 | ± | 8.44 | 30.9 | ± | 4.76 |
| **CD4+ Effector memory T cell** | 19.3 | ± | 7.78 | 23.5 | ± | 13.5 | 18.5 | ± | 11.3 | 12.2 | ± | 8.94 | 11.5 | ± | 10.4 |
| **NEDA** | 18.3 | ± | 8.52 | 24.3 | ± | 14.3 | 18.9 | ± | 11.6 | 10.3 | ± | 7.82 | 8.33 | ± | 8.13 |
| **ODA** | 21.3 | ± | 6.37 | 23.4 | ± | 13.0 | 19.0 | ± | 11.6 | 17.9 | ± | 10.4 | 19.6 | ± | 12.5 |
| **Th1 Effector memory** | 38.5 | ± | 9.52 | 36.7 | ± | 11.3 | 34.2 | ± | 15.1 | 35.0 | ± | 11.5 | 35.3 | ± | 8.43 |
| **NEDA** | 36.3 | ± | 8.55 | 35.1 | ± | 12.1 | 32.3 | ± | 16.2 | 35.0 | ± | 12.8 | 35.0 | ± | 8.27 |
| **ODA** | 43.9 | ± | 11.2 | 40.1 | ± | 9.99 | 36.2 | ± | 12.0 | 34.8 | ± | 9.86 | 34.6 | ± | 9.54 |
| **Th2 Effector memory** | 10.0 | ± | 4.46 | 10.4 | ± | 5.51 | 13.0 | ± | 10.6 | 12.9 | ± | 7.79 | 16.0 | ± | 8.72 |
| **NEDA** | 10.4 | ± | 4.55 | 11.1 | ± | 6.13 | 14.1 | ± | 11.9 | 14.5 | ± | 8.77 | 17.8 | ± | 9.64 |
| **ODA** | 9.32 | ± | 4.95 | 7.83 | ± | 2.54 | 8.00 | ± | 3.49 | 8.95 | ± | 3.68 | 11.1 | ± | 4.46 |
| **Th17 Effector memory** | 15.8 | ± | 6.49 | 16.6 | ± | 6.39 | 23.3 | ± | 19.1 | 21.9 | ± | 9.35 | 23.6 | ± | 8.45 |
| **NEDA** | 16.9 | ± | 6.36 | 17.0 | ± | 6.74 | 26.5 | ± | 22.2 | 22.3 | ± | 10.2 | 24.2 | ± | 8.37 |
| **ODA** | 13.2 | ± | 7.14 | 14.7 | ± | 6.11 | 17.8 | ± | 6.35 | 21.0 | ± | 8.76 | 23.4 | ± | 9.79 |
| **Th1/Th17Effector memory** | 35.7 | ± | 8.88 | 36.3 | ± | 9.55 | 29.5 | ± | 12.6 | 30.3 | ± | 10.8 | 25.1 | ± | 9.63 |
| **NEDA** | 36.6 | ± | 9.80 | 36.5 | ± | 11.0 | 26.1 | ± | 12.9 | 27.4 | ± | 11.5 | 22.8 | ± | 10.9 |
| **ODA** | 33.6 | ± | 8.52 | 37.3 | ± | 4.95 | 38.0 | ± | 6.74 | 35.3 | ± | 8.44 | 30.9 | ± | 4.76 |
| **CD4+ T_EMRA_** | 3.81 | ± | 6.99 | 3.27 | ± | 3.69 | 5.58 | ± | 12.7 | 1.50 | ± | 1.62 | 1.27 | ± | 1.31 |
| **NEDA** | 2.72 | ± | 3.95 | 3.10 | ± | 2.51 | 7.58 | ± | 15.1 | 1.64 | ± | 1.92 | 1.17 | ± | 1.47 |
| **ODA** | 6.78 | ± | 12.1 | 4.03 | ± | 6.16 | 1.18 | ± | 0.77 | 1.22 | ± | 0.68 | 1.58 | ± | 0.99 |
| **T regulatory subsets** |  |  |  |  |  |  |  |  |  |  |  |  |  |  |  |
| **Memory Treg** | 8.60 | ± | 2.22 | 8.51 | ± | 1.85 | 8.09 | ± | 1.58 | 7.86 | ± | 2.46 | 7.87 | ± | 1.61 |
| **NEDA** | 4.28 | ± | 1.52 | 4.37 | ± | 1.38 | 3.39 | ± | 1.30 | 3.27 | ± | 1.75 | 2.81 | ± | 1.13 |
| **ODA** | 5.03 | ± | 1.50 | 4.99 | ± | 2.46 | 4.28 | ± | 1.96 | 4.12 | ± | 1.90 | 3.22 | ± | 1.41 |
| **Activated memory Treg** | 39.3 | ± | 11.5 | 39.4 | ± | 9.09 | 38.3 | ± | 8.99 | 37.1 | ± | 10.6 | 36.7 | ± | 13.5 |
| **NEDA** | 37.6 | ± | 10.8 | 36.7 | ± | 9.33 | 36.3 | ± | 9.53 | 35.8 | ± | 9.53 | 32.4 | ± | 9.99 |
| **ODA** | 42.5 | ± | 14.5 | 44.1 | ± | 6.54 | 40.9 | ± | 6.68 | 35.2 | ± | 5.25 | 44.2 | ± | 17.4 |
| **CD8+ T cell subsets** |  |  |  |  |  |  |  |  |  |  |  |  |  |  |  |
| **CD8 lymphocytes** | 36.4 | ± | 9.95 | 35.8 | ± | 8.62 | 33.5 | ± | 8.02 | 31.8 | ± | 7.45 | 30.0 | ± | 7.64 |
| **CD8+ Naïve T cell** | 42.7 | ± | 20.6 | 36.7 | ± | 19.0 | 45.5 | ± | 23.6 | 52.8 | ± | 24.5 | 56.3 | ± | 22.4 |
| **NEDA** | 45.0 | ± | 23.0 | 37.3 | ± | 21.0 | 45.6 | ± | 23.7 | 54.7 | ± | 23.1 | 60.9 | ± | 21.6 |
| **ODA** | 36.1 | ± | 15.2 | 33.7 | ± | 15.7 | 38.6 | ± | 20.6 | 41.7 | ± | 24.3 | 44.0 | ± | 23.5 |
| **CD8+ Central memory T cell** | 10.2 | ± | 9.69 | 8.31 | ± | 6.01 | 5.69 | ± | 3.66 | 4.73 | ± | 3.16 | 3.83 | ± | 2.30 |
| **NEDA** | 7.98 | ± | 5.12 | 7.05 | ± | 4.26 | 4.77 | ± | 2.98 | 3.69 | ± | 1.41 | 3.65 | ± | 1.96 |
| **ODA** | 16.5 | ± | 16.0 | 11.9 | ± | 8.86 | 8.60 | ± | 4.02 | 7.73 | ± | 4.59 | 4.37 | ± | 3.32 |
| **CD8+ Effector memory T cell** | 22.5 | ± | 11.9 | 27.9 | ± | 12.0 | 21.9 | ± | 11.5 | 17.9 | ± | 11.2 | 13.4 | ± | 11.4 |
| **NEDA** | 22.9 | ± | 13.8 | 28.0 | ± | 13.6 | 22.4 | ± | 12.5 | 16.9 | ± | 11.4 | 11.6 | ± | 10.5 |
| **ODA** | 22.0 | ± | 7.42 | 26.9 | ± | 9.18 | 23.1 | ± | 8.79 | 22.4 | ± | 9.90 | 18.9 | ± | 13.7 |
| **CD8+ T_EMRA_** | 24.6 | ± | 15.0 | 27.1 | ± | 13.7 | 27.0 | ± | 15.5 | 24.7 | ± | 15.0 | 26.4 | ± | 16.3 |
| **NEDA** | 24.2 | ± | 15.8 | 27.8 | ± | 14.8 | 27.2 | ± | 16.6 | 24.8 | ± | 15.1 | 23.8 | ± | 14.7 |
| **ODA** | 25.4 | ± | 15.5 | 27.5 | ± | 11.7 | 29.7 | ± | 12.1 | 28.1 | ± | 14.4 | 32.8 | ± | 20.9 |

Data shown in blue indicate statistically significant differences compared to baseline levels (p<0.05).

Th1/Th17= Th1-like Th17 cells
