# Supplementary material for: Optimal response to dimethyl fumarate is mediated by a reduction of Th1‐like Th17 cells after 3 months of treatment
Source: CNS Neurosci Ther. 2019 May 7;25(9):995–1005. doi: 10.1111/cns.13142 (PMC6698982; doi:10.1111/cns.13142)
Supplement: Supplementary file 7 [file CNS-25-995-s007.docx]

**Supplementary table 4.** Absolute counts of B lymphocytes, NK cells, monocyte and DC subpopulations in RRMS patients under dimethyl fumarate treatment during 12-months follow-up.

|  | **Baseline** | | | **+1month** | | | **+3month** | | | **+6month** | | | **+12month** | | |
| --- | --- | --- | --- | --- | --- | --- | --- | --- | --- | --- | --- | --- | --- | --- | --- |
|  | **(cells/µl)** | | | **(cells/µl)** | | | **(cells/µl)** | | | **(cells/µl)** | | | **(cells/µl)** | | |
| **B cell subsets** |  |  |  |  |  |  |  |  |  |  |  |  |  |  |  |
| **CD19+ cells** | 156 | ± | 17 | 123 | ± | 15 | 132 | ± | 22 | 113 | ± | 12 | 118 | ± | 24 |
| **Naïve B cells** | 93 | ± | 2 | 73 | ± | 2 | 89 | ± | 2 | 82 | ± | 1 | 92 | ± | 2 |
| **Transitional B cells** | 6 | ± | 0 | 6 | ± | 0 | 11 | ± | 0 | 11 | ± | 0 | 12 | ± | 0 |
| **Pre-switched memory B cells** | 30 | ± | 1 | 22 | ± | 1 | 21 | ± | 1 | 15 | ± | 1 | 12 | ± | 1 |
| **Switched memory B cells** | 29 | ± | 1 | 24 | ± | 1 | 18 | ± | 2 | 13 | ± | 1 | 11 | ± | 1 |
| **Plasma cells** | 2 | ± | 0 | 2 | ± | 0 | 1 | ± | 0 | 1 | ± | 0 | 1 | ± | 0 |
| **Double negative B cells** | 5 | ± | 0 | 4 | ± | 0 | 3 | ± | 0 | 3 | ± | 0 | 3 | ± | 0 |
| **Natural killer (NK) cell subsets** |  |  |  |  |  |  |  |  |  |  |  |  |  |  |  |
| **NK cells** | 138 | ± | 3 | 138 | ± | 7 | 150 | ± | 5 | 116 | ± | 7 | 131 | ± | 6 |
| **CD56bright CD16- NK cells** | 13 | ± | 0 | 12 | ± | 0 | 14 | ± | 0 | 13 | ± | 0 | 17 | ± | 0 |
| **CD56dim CD16+ NK cells** | 113 | ± | 0 | 113 | ± | 1 | 120 | ± | 1 | 87 | ± | 1 | 100 | ± | 1 |
| **Monocyte subsets** |  |  |  |  |  |  |  |  |  |  |  |  |  |  |  |
| **Monocytes** | 543 | ± | 10 | 612 | ± | 16 | 560 | ± | 13 | 543 | ± | 16 | 554 | ± | 15 |
| **Classical Monocytes** | 498 | ± | 1 | 556 | ± | 2 | 519 | ± | 1 | 506 | ± | 1 | 521 | ± | 1 |
| **Non-classical Monocytes** | 45 | ± | 1 | 57 | ± | 2 | 42 | ± | 1 | 38 | ± | 1 | 34 | ± | 1 |
| **Dendritic cells (DC) subsets** |  |  |  |  |  |  |  |  |  |  |  |  |  |  |  |
| **DC** | 179 | ± | 4 | 165 | ± | 7 | 158 | ± | 5 | 156 | ± | 9 | 156 | ± | 6 |
| **Myeloid DC** | 92 | ± | 1 | 79 | ± | 1 | 90 | ± | 1 | 82 | ± | 2 | 82 | ± | 1 |
| **Plasmacytoid DC** | 65 | ± | 1 | 64 | ± | 1 | 49 | ± | 1 | 54 | ± | 2 | 43 | ± | 1 |

Data shown in blue indicate statistically significant differences compared to baseline levels (p<0.05).
