# Supplementary material for: Optimal response to dimethyl fumarate is mediated by a reduction of Th1‐like Th17 cells after 3 months of treatment
Source: CNS Neurosci Ther. 2019 May 7;25(9):995–1005. doi: 10.1111/cns.13142 (PMC6698982; doi:10.1111/cns.13142)
Supplement: Supplementary file 8 [file CNS-25-995-s008.docx]

**Supplementary table 5.** Percentages of B lymphocytes, NK cells, monocyte and DC subpopulations in RRMS patients under dimethyl fumarate treatment during 12-months follow-up.

|  | **Baseline** | | | **+1month** | | | **+3month** | | | **+6month** | | | **+12month** | | |
| --- | --- | --- | --- | --- | --- | --- | --- | --- | --- | --- | --- | --- | --- | --- | --- |
|  | **(%)** | | | **(%)** | | | **(%)** | | | **(%)** | | | **(%)** | | |
| **B cell subsets** |  |  |  |  |  |  |  |  |  |  |  |  |  |  |  |
| **CD19+ cells** | 10.1 | ± | 2.90 | 8.64 | ± | 2.82 | 9.45 | ± | 3.23 | 10.3 | ± | 2.74 | 10.7 | ± | 3.65 |
| **Naïve B cells** | 59.6 | ± | 12.2 | 59.6 | ± | 11.8 | 67.4 | ± | 10.9 | 72.4 | ± | 10.3 | 77.7 | ± | 9.02 |
| **NEDA** | 59.1 | ± | 13.3 | 57.4 | ± | 11.4 | 67.1 | ± | 10.4 | 73.2 | ± | 10.1 | 76.9 | ± | 9.02 |
| **ODA** | 59.8 | ± | 12.7 | 62.1 | ± | 10.9 | 64.6 | ± | 8.02 | 66.9 | ± | 7.39 | 76.9 | ± | 9.24 |
| **Transitional B cells** | 6.89 | ± | 4.25 | 7.73 | ± | 3.45 | 12.6 | ± | 6.31 | 12.9 | ± | 7.54 | 13.6 | ± | 6.34 |
| **NEDA** | 7.15 | ± | 4.58 | 7.88 | ± | 3.84 | 12.5 | ± | 6.93 | 13.0 | ± | 6.91 | 12.5 | ± | 4.64 |
| **ODA** | 7.37 | ± | 2.69 | 7.60 | ± | 2.83 | 11.1 | ± | 2.56 | 13.7 | ± | 9.71 | 17.1 | ± | 9.20 |
| **Pre-switched memory B cells** | 19.2 | ± | 8.99 | 18.3 | ± | 7.66 | 16.2 | ± | 6.72 | 13.3 | ± | 5.90 | 10.6 | ± | 5.48 |
| **NEDA** | 21.0 | ± | 9.48 | 20.1 | ± | 6.93 | 17.3 | ± | 6.91 | 13.7 | ± | 6.13 | 11.6 | ± | 5.99 |
| **ODA** | 15.6 | ± | 8.59 | 15.0 | ± | 7.42 | 14.6 | ± | 5.07 | 14.0 | ± | 5.84 | 9.70 | ± | 4.49 |
| **Switched memory B cells** | 18.3 | ± | 7.82 | 19.2 | ± | 7.96 | 13.8 | ± | 7.30 | 11.4 | ± | 6.92 | 9.38 | ± | 4.72 |
| **NEDA** | 17.4 | ± | 8.80 | 19.7 | ± | 8.77 | 13.1 | ± | 6.43 | 10.8 | ± | 6.18 | 9.24 | ± | 4.46 |
| **ODA** | 21.2 | ± | 6.32 | 20.0 | ± | 6.80 | 17.6 | ± | 8.29 | 14.7 | ± | 8.07 | 11.1 | ± | 5.20 |
| **Plasma cells** | 1.01 | ± | 1.28 | 1.59 | ± | 1.21 | 0.47 | ± | 0.33 | 0.64 | ± | 0.51 | 0.57 | ± | 0.36 |
| **NEDA** | 1.12 | ± | 1.40 | 1.71 | ± | 1.32 | 0.37 | ± | 0.11 | 0.60 | ± | 0.52 | 0.52 | ± | 0.36 |
| **ODA** | 0.50 | ± | 0.16 | 1.46 | ± | 1.25 | 0.65 | ± | 0.51 | 0.55 | ± | 0.44 | 0.63 | ± | 0.39 |
| **Double negative B cells** | 3.00 | ± | 1.49 | 2.88 | ± | 1.08 | 2.65 | ± | 1.37 | 2.39 | ± | 1.29 | 2.38 | ± | 1.56 |
| **NEDA** | 2.55 | ± | 1.40 | 2.78 | ± | 1.12 | 2.52 | ± | 1.38 | 2.24 | ± | 1.26 | 2.24 | ± | 1.30 |
| **ODA** | 3.45 | ± | 1.45 | 2.87 | ± | 1.16 | 3.23 | ± | 1.24 | 2.72 | ± | 0.79 | 2.27 | ± | 0.76 |
| **Natural killer (NK) cell subsets** |  |  |  |  |  |  |  |  |  |  |  |  |  |  |  |
| **NK cells** | 18.8 | ± | 4.97 | 17.0 | ± | 7.77 | 19.3 | ± | 7.32 | 16.2 | ± | 6.85 | 18.3 | ± | 6.51 |
| **CD56bright CD16- NK cells** | 9.31 | ± | 6.05 | 9.01 | ± | 5.24 | 9.46 | ± | 6.92 | 11.5 | ± | 7.23 | 12.7 | ± | 7.06 |
| **NEDA** | 9.73 | ± | 6.96 | 8.15 | ± | 4.85 | 9.33 | ± | 7.92 | 10.5 | ± | 6.22 | 12.4 | ± | 5.87 |
| **ODA** | 7.98 | ± | 3.68 | 11.4 | ± | 6.66 | 8.54 | ± | 3.57 | 10.5 | ± | 5.11 | 12.4 | ± | 10.6 |
| **CD56dim CD16+ NK cells** | 81.9 | ± | 10.4 | 82.0 | ± | 9.63 | 80.1 | ± | 13.2 | 74.6 | ± | 14.5 | 76.8 | ± | 13.0 |
| **NEDA** | 80.7 | ± | 11.9 | 83.5 | ± | 9.18 | 79.9 | ± | 15.2 | 74.8 | ± | 14.5 | 76.6 | ± | 14.2 |
| **ODA** | 83.3 | ± | 6.02 | 79.0 | ± | 9.40 | 83.5 | ± | 4.77 | 78.9 | ± | 6.13 | 77.6 | ± | 9.32 |
| **Monocyte subsets** |  |  |  |  |  |  |  |  |  |  |  |  |  |  |  |
| **Monocytes** | 73.9 | ± | 18.8 | 75.3 | ± | 17.1 | 71.9 | ± | 18.8 | 75.9 | ± | 17.2 | 77.8 | ± | 16.1 |
| **Classical Monocytes** | 91.7 | ± | 5.92 | 90.9 | ± | 10.2 | 92.6 | ± | 5.06 | 93.2 | ± | 3.70 | 94.0 | ± | 3.80 |
| **Non-classical Monocytes** | 8.38 | ± | 5.94 | 9.24 | ± | 10.2 | 7.53 | ± | 5.22 | 6.94 | ± | 3.76 | 6.09 | ± | 3.83 |
| **Dendritic cells (DC) subsets** |  |  |  |  |  |  |  |  |  |  |  |  |  |  |  |
| **DC** | 48.9 | ± | 13.4 | 40.6 | ± | 14.5 | 40.5 | ± | 15.3 | 43.6 | ± | 19.0 | 43.8 | ± | 13.3 |
| **Myeloid DC** | 51.4 | ± | 15.9 | 48.0 | ± | 17.9 | 57.4 | ± | 13.4 | 52.6 | ± | 17.2 | 52.5 | ± | 11.7 |
| **Plasmacytoid DC** | 36.2 | ± | 14.4 | 38.8 | ± | 20.4 | 31.4 | ± | 10.5 | 34.7 | ± | 17.2 | 27.4 | ± | 12.8 |

Data shown in blue indicate statistically significant differences compared to baseline levels (p<0.05).

Data for NEDA and ODA patients are also exhibited for subsets showing statistically significant differences (p<0.05).
